# Vitamin B12, Homocysteine and Cognitive Impairment in Elderly Patients with Type 2 Diabetes

**DOI:** 10.3390/jcm15155935

**Published:** 2026-07-29

**Authors:** Malgorzata Gorska-Ciebiada, Maciej Ciebiada

**Affiliations:** 1 Department of Propaedeutics of Lifestyle Diseases, Medical University of Lodz, 90-251 Lodz, Poland; 2 Department of General and Oncological Pulmonology, Medical University of Lodz, 90-549 Lodz, Poland

**Keywords:** mild cognitive impairment, vitamin B12, homocysteine, elderly, type 2 diabetes

## Abstract

**Background:** Type 2 diabetes (T2DM) increases the risk of mild cognitive impairment (MCI); however, the mechanisms of this process have not yet been fully identified. The aim of the study was to determine the levels of vitamin B12 and homocysteine in elderly diabetic patients, with and without cognitive impairment, and identify the risk factors associated with MCI in this group. **Methods:** A total of 385 elderly diabetic patients were screened for MCI (using the Montreal Cognitive Assessment). Several clinical and biochemical data were recorded. **Results:** MCI subjects had lower vitamin B12 levels (251 ± 36.9 pg/mL) and higher homocysteine concentrations (15.9 ± 4 ng/mL) compared to controls (vitamin B12: 339.9 ± 40.7 pg/mL; homocysteine: 11.4 ± 3.1 ng/mL). In MCI subjects, vitamin B12 levels were negatively correlated with homocysteine or HbA_1c_ levels and positively correlated with MoCA scores. The univariate logistic regression showed factors associated with MCI in elderly diabetic patients were the following: older age and fewer years of education, longer history of diabetes, a higher number of co-morbidities, higher levels of HbA_1c_, triglycerides and homocysteine, lower levels of vitamin B12, presence of cardiovascular disease, hypertension, hyperlipidemia, retinopathy, and nephropathy. Independent factors associated with MCI evaluated in the multivariate model included lower levels of vitamin B12, higher levels of triglycerides, and a higher number of co-morbidities and fewer years of formal education. **Conclusions:** Vitamin B12 is associated with MCI in elderly diabetic patients. The role of homocysteine and vitamin B12 in the common pathogenesis of cognitive impairment and diabetes requires future prospective and more extensive studies.

## 1. Introduction

The prevalence of type 2 diabetes (T2DM) is increasing rapidly, becoming a major global health issue. This number is expected to grow from 588.7 million in 2024 to 852.5 million by 2050 [1]. This serious disease increases the risk of several comorbidities, one of which is cognitive impairment and dementia [2]. Adults with T2DM are approximately 1.6 times more likely to develop dementia than individuals without diabetes [2], which might affect self-care management, worsen diabetic control and increase mortality [3]. With a growing aging population, cognitive impairment is becoming a more prevalent condition in the elderly [4]. Mild cognitive impairment (MCI) is distinguished by a reduction in one or more cognitive abilities in neurocognitive testing, including memory, language, calculation, emotions, attention, orientation, and executive function [5]. Although this condition does not seriously affect activities of daily life, it may lead to higher rates of disability and mortality, thus constituting a considerable public health burden. MCI is considered a phase occurring between typical cognitive well-being and dementia [5,6]. It has been indicated that even 15% of patients with cognitive impairment aged over 65 years develop dementia in two years [7].

Age-related deterioration in cognition is typically connected to alterations in both the structure and functioning of the brain, such as decline in gray and white matter volume, decreased synaptic plasticity, impaired large-scale functional connectivity, and lower neurotransmitter levels [8]. Many studies indicate that cerebral microvascular dysfunction is crucial in the development of dementia in older people [9]. In a diabetic brain, microcirculatory damage is evident through the presence of hyperintensities in white matter and lacunes that are thought to be of vascular origin. This condition also includes cerebral microbleeds, perivascular spaces, overall cerebral atrophy, and small infarcts [10]. These characteristics of cerebral small vessel disease are connected to increased oxidative stress, inflammatory response and changes in the permeability of the blood–brain barrier. In diabetes, factors that contribute to microvascular dysfunction include hyperglycemia, insulin resistance, hypertension and arterial stiffness [10].

The mechanisms underlying type 2 diabetes-related cognitive impairment seem to be multifactorial; however, they have not yet been fully identified. Vitamin B12 deficiency is associated with many neuropsychiatric conditions and is mentioned to be a reversible factor contributing to dementia [11]. Vitamin B12, also known as cobalamin, is a vitamin that dissolves in water and is essential for the modulation of gene expression, DNA synthesis, and red blood cell production [12]. It is also a cofactor for cytosolic methionine synthase and the mitochondrial methylmalonyl coenzyme A. Cobalamin deficiency leads to accumulation of neurotoxic substances, e.g., homocysteine and methylmalonic acid. The disturbed function of methionine synthase causes a reduction in metabolites of methionine, which plays a crucial role in the production of neurotransmitters, phospholipids and myelin formation [13]. Nevertheless, hyperhomocysteinemia, resulting from vitamin B12 deficiency, has been found to elevate inflammation, enhance oxidative stress, and contribute to a range of illnesses such as cardiovascular disease, stroke, and dementia [14,15,16]. In general, vitamin B12 levels decrease with age, and various conditions, such as gastritis, malabsorption, bacterial overgrowth, poorer nutrition, and regular use of drugs, affect the release of vitamin B12 from food sources [17]. Although the elderly population has a higher risk of vitamin B12 deficiency and hyperhomocysteinemia, the relationship between these conditions and cognitive function has not yet been clearly defined. Some research findings have indicated that vitamin B12 plays a significant role in decreasing the risk of cognitive decline, which is linked to enhanced cognitive abilities in elderly individuals [18,19]. Some studies have indicated an association between low vitamin B12 levels and poor cognition or dementia [20,21]. Others, however, do not confirm this observation [22].

Furthermore, the meta-analysis showed that vitamin B supplementation effectively lowered homocysteine levels without any improvement in cognition in elderly individuals diagnosed with Alzheimer’s disease or dementia [23].

Considering the increasing number of elderly individuals with T2DM, it is important to understand the changes in cognitive health and recognize related risk factors. It is crucial for early identification and prevention of neurodegenerative disorders linked to aging. While the association between vitamin B12 or homocysteine levels and cognitive status has been previously documented in the general population, comprehensive data focusing specifically on older adults with diabetes are limited. This is a critical gap, as diabetes independently accelerates cognitive decline, and common diabetic management can alter vitamin metabolic pathways. To address this gap, this study offers a novel perspective by examining the levels of both vitamin B12 and homocysteine specifically in the elderly Polish outpatient diabetic population, comparing those with and without cognitive impairment. Furthermore, apart from assessing single correlations, this study aims to identify the comprehensive, multi-factorial risk factors associated with MCI within this particular clinical cohort. Thus, we aim to clarify how metabolic markers interact with cognitive health in the diabetic aging population, potentially highlighting areas for early screening.

Therefore, the aims of the study were twofold: firstly, to determine the levels of vitamin B12 and homocysteine in elderly diabetic patients with and without cognitive impairment, and secondly, to identify the risk factors associated with MCI in this group.

## 2. Materials and Methods

### 2.1. Study Design

This cross-sectional study was conducted in the Specialist Outpatient Clinic of Diabetes, the N. Barlicki Memorial Teaching Hospital No. 1 of the Medical University of Lodz. The participants were briefly screened for recruitment and gave informed consent to enter the study. After the collection of fasting blood samples, the patients received a snack to make sure they were not experiencing hypoglycemia during psychological evaluation. In the next stage, in a private and discreet environment, a standard interview was taken, and physical examination and neuropsychological assessment were also conducted. Finally, a total of 385 elderly patients with type 2 diabetes were recruited. The participants were selected into two groups: one including 126 subjects diagnosed with MCI and the other including 259 patients evaluated as cognitively normal.

### 2.2. Participant Selection

The study participants were recruited based on predefined eligibility criteria. The inclusion criteria were the following: age 65 years or older, confirmed diagnosis of T2DM for at least one year and ability to understand and cooperate with study procedures.

The study excluded subjects with a history of any diseases or medications that could confound the results of vitamin B12 and homocysteine levels. The exclusion criteria were as follows: gastrointestinal disorders, e.g., malabsorption or chronic gastritis, ulcerative colitis, Crohn’s disease, celiac disease, bariatric surgeries and ileal resection, alcohol or substance abuse, use of drugs that affect vitamin B12 metabolism, such as colchicine, chloramphenicol, cimetidine, neomycin, phenytoin, methyldopa, long-term use of medications affecting secretion of gastric acid or pH (proton pump inhibitors, H2 receptor antagonists), pernicious anemia, vegan or vegetarian diet, vitamin B12 supplementation within the past three months, severe illness, e.g., an active infection, sepsis, malignancy. The study also excluded participants who had a head injury, suffered from depression or dementia, had other neurological or psychiatric conditions, had taken cognition-impairing treatment in the last three months or had severe hearing, visual or motor coordination impairment.

### 2.3. Assessment of Cognitive Function

First, the Montreal Cognitive Assessment (MoCA) tool was used in all the subjects. It comprises eight domains: visuospatial/executive reasoning, memory, naming, abstraction, attention, language, and orientation skills [24]. The MoCA test has been shown to be sensitive in distinguishing patients with MCI from cognitively normal individuals, and it has been regarded as the best screening tool in the elderly and diabetic patients [25,26]. The maximum possible total score in the MoCA test is 30 points, and a cut-off of ≥26 is considered normal. It also takes into account less than 12 years of education by adding 1 point to the final score. In line with epidemiological studies, MoCA test scores below 19 points were considered to indicate ‘dementia’, and individuals with such scores were excluded from the study and referred to a psychiatrist for further assessment.

In this study, MCI diagnosis was established according to the diagnostic criteria proposed by the 2006 European Alzheimer’s Disease Consortium [27]. These procedure included the following items: cognitive complaints reported by the patient and/or their family, cognitive decline reported by the patient or informant within the past year; objective evidence of cognitive impairment, i.e., neuropsychological test scores below the norm for age and education, no impact on daily functioning (maintaining independence in activities of daily living although with some difficulties when performing more complex tasks), absence of dementia (not meeting the Diagnostic and Statistical Manual of Mental Disorders-IV (DSM-IV) criteria for dementia syndromes) [27]. In order to reduce educational bias and ensure diagnostic accuracy, the MoCA score was integrated into a comprehensive clinical evaluation. The diagnosis of MCI required both subjective cognitive complaints reported by the subject during the interview and preserved daily functioning (in our study, measured by Katz Basic Activities (BADL) and Lawton Instrumental Activities of Daily Living (IADL).

### 2.4. Sample Collection and Biochemical Analysis

After overnight fasting, samples of peripheral venous blood (a total of 10 mL) were collected from each participant under aseptic conditions for various biochemical tests. Total cholesterol, low-density lipoprotein cholesterol (LDL), high-density lipoprotein cholesterol (HDL), triglycerides (TG), fasting blood glucose (FBG) and glycosylated hemoglobin (HbA_1c_) levels were measured in a certified centralized laboratory. The blood samples were centrifuged within 30 min following collection and subsequently stored at a temperature of −80 °C. Measurements were performed after all the subjects had been enrolled in the study. Serum vitamin B12 (Vitamin B12 ELISA kit, ab287826, BioVision, an Abcam company, Cambridge, UK) and homocysteine levels (General Hcy/Homocysteine ELISA kit, E0772Ge, EIAab, Wuhan, China) were determined according to the manufacturer’s instructions. The detection ranges of the enzyme-linked immunosorbent assays (ELISA) for vitamin B12 and homocysteine were 50–500 pg/mL and 0.78–50 ng/mL, respectively. For optimal performance, all samples and controls were assayed in duplicate. Absorbance was read at 450 nm after adding the stop solution. Minimum detectable concentrations were 25 pg/mL for vitamin B12 and 0.38 ng/mL for homocysteine, respectively, with an overall intra-assay coefficient of variation (CV) of < 8% and an inter-assay CV of <10%.

### 2.5. Assessment of Demographic and Other Variables

Clinical and demographic data were collected through structured interviews and medical record reviews. The following variables were considered: age, gender, educational level, lifestyle factors (including smoking and physical activity), duration of diabetes, medication use, hypertension, hyperlipidemia, retinopathy, nephropathy, neuropathy, stroke, cardiovascular disease, and comorbidities. Height and weight of all the subjects were measured by a trained nurse using a calibrated stadiometer and digital scale, and body mass index (BMI = weight in kg/height in m^2^) was calculated. Blood pressure was measured with a validated mercury sphygmomanometer using the right arm, in a seated position in accordance with standard operating procedures. Hypertension was diagnosed based on two blood pressure measurements of ≥140/90 mmHg or a prior report of active hypertension and current use of antihypertensive medications. Hyperlipidemia was determined based on either a history of the use of any lipid-lowering drug, or untreated levels of triglycerides over 1.7 mmol/L or/and serum LDL cholesterol levels over 2.6 mmol/L.

### 2.6. Ethical Approval

The study was conducted in full compliance with the guidelines of good clinical practice of the World Medical Association (WMA) Declaration of Helsinki. The purpose, nature, and potential risks of the experiments were fully explained to the subjects before admission, and prior to enrollment, all the subjects gave informed consent. Each patient was assigned a number by which they were identified to ensure privacy. The study was approved by the institutional medical ethical committee (Medical University of Lodz, Approval No. RNN/420/13/KB).

### 2.7. Statistical Analysis

Statistical analyses were performed using Statistica 13.1 (StatSoft, Kraków, Poland). The continuous variables were presented as mean and standard deviation (SD), whereas the categorical variables were reported as absolute frequencies and percentages. An a priori power analysis was performed using G*Power software (version 3.1.9.7; Heinrich Heine University Dusseldorf, Düsseldorf, Germany) to determine the required sample size for a two-tailed independent samples test. Setting the significance at α = 0.05, targeting a statistical power of 0.95, and considering a medium effect size d = 0.50, with an allocation ratio of 2:1, the minimum required total sample size was calculated to be 236 participants (N1 = 79) and (N2 = 157). The final sample obtained for this study exceeded this requirement, consisting of 385 individuals, with 126 in Group1 and 259 in Group 2.

The distribution of data was examined using the Shapiro–Wilk Test. An independent *t*-test was used to compare normally distributed continuous variables. The Mann–Whitney U test was performed for skewed data. Categorical data were analyzed using the chi-square (χ^2^) test. The relationships were assessed using Pearson’s correlation analysis for normally distributed variables, or Spearman’s rank correlation for non-normally distributed variables. A simple logistic regression model was developed to select so-called independent factors that increase the selection risk of MCI in elderly patients with T2DM. To evaluate the associations of vitamin B12 or homocysteine levels with MCI, a multivariable logistic regression model was developed to assess whether circulating mediators were independently associated with MCI. To “optimize” the multivariable model, a stepwise approach was applied (backward elimination based on the Wald criteria). Odds ratios (OR) were computed and presented with the 95% confidence interval (CI). A *p*-value of <0.05 was considered statistically significant.

## 3. Results

### 3.1. Clinical and Laboratory Characteristics of T2DM Patients

The characteristics related to the demographics, clinical aspects, and laboratory findings of the study group are presented in Table 1. The results of the Mann–Whitney U test or T-test showed that patients with MCI were older, had a lower level of education, had a longer duration of diabetes, a higher number of comorbidities, increased levels of HbA_1c_, total cholesterol and triglycerides, lower levels of HDL cholesterol and lower MoCA scores (Table 1). Furthermore, the χ^2^ test showed that subjects with MCI were significantly more likely to be diagnosed with CVD, hypertension, hyperlipidemia, retinopathy and nephropathy (Table 1). Lastly, no significant differences were found between the groups in terms of gender, smoking habits, physical activity, stroke, diabetic polyneuropathy, and type of treatment, BMI, levels of LDL cholesterol, FBG and systolic/diastolic blood pressure (*p* > 0.05).

### 3.2. Serum Levels of Vitamin B12 and Homocysteine in the Elderly T2DM Patients with MCI and Those Without MCI

**Figure 1:**
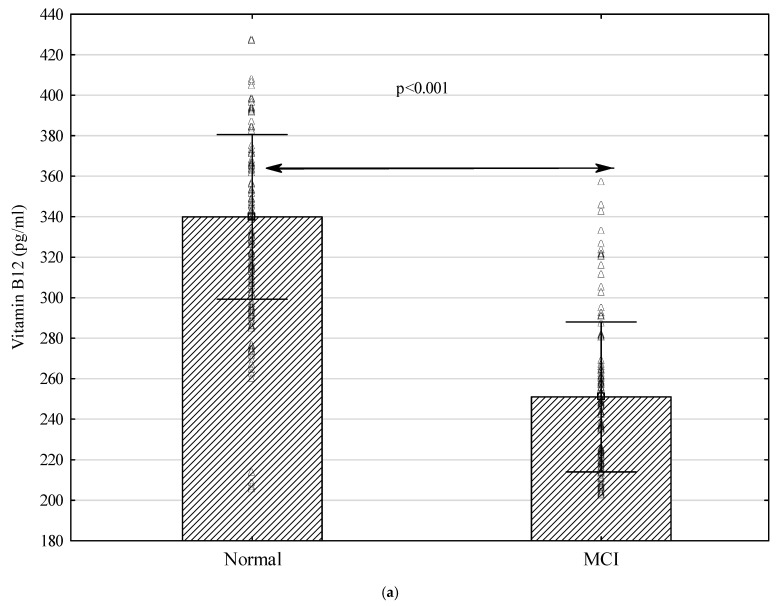

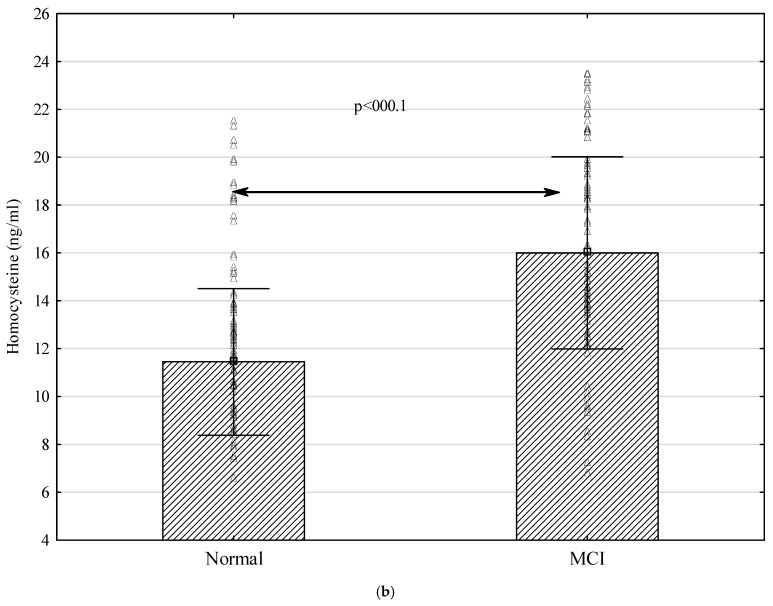
Serum levels of Vitamin B12 (pg/mL) (**a**) and Homocysteine (ng/mL) (**b**) in the elderly T2DM patients with mild cognitive impairment (MCI) and those without MCI (Normal).

**Figure 2:**
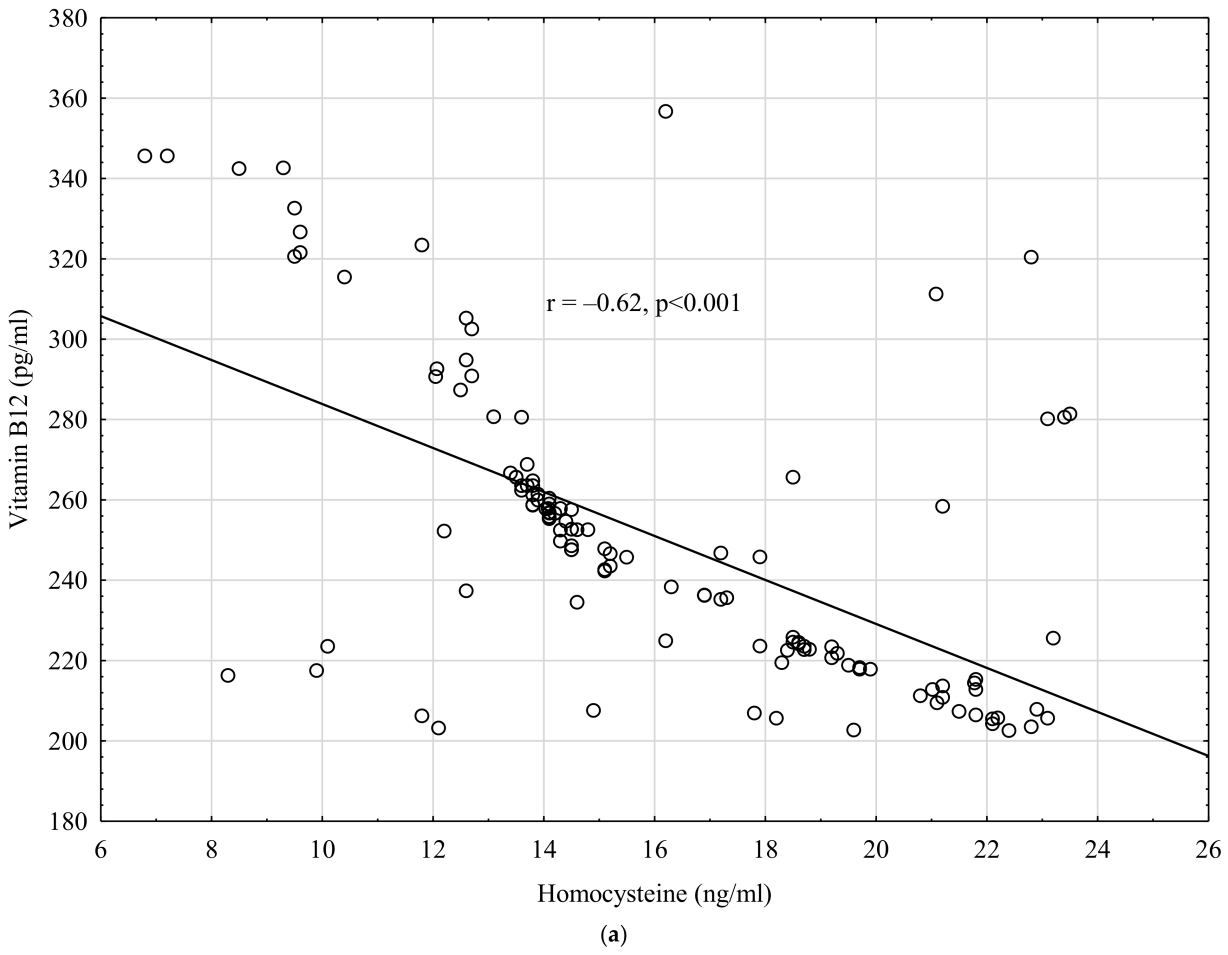

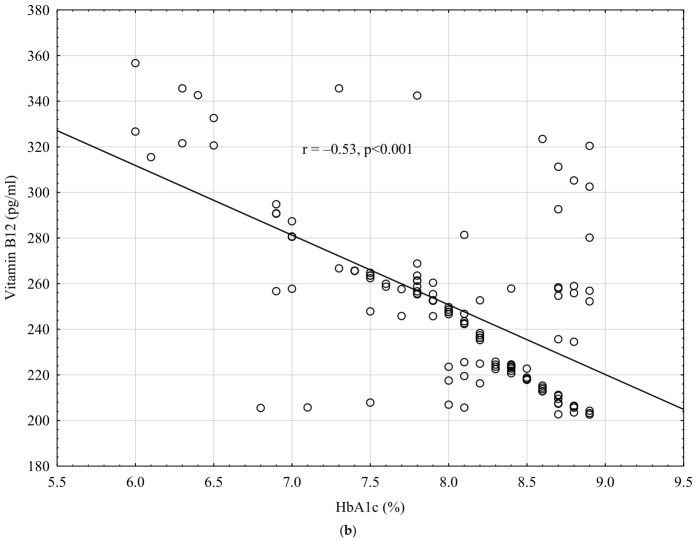

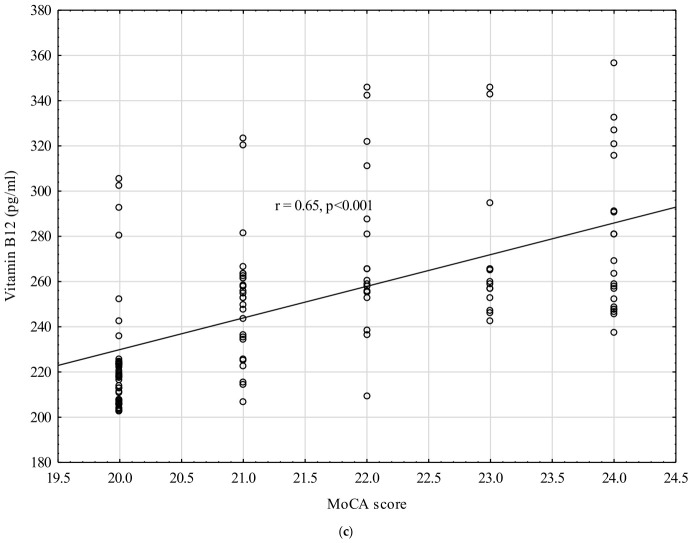

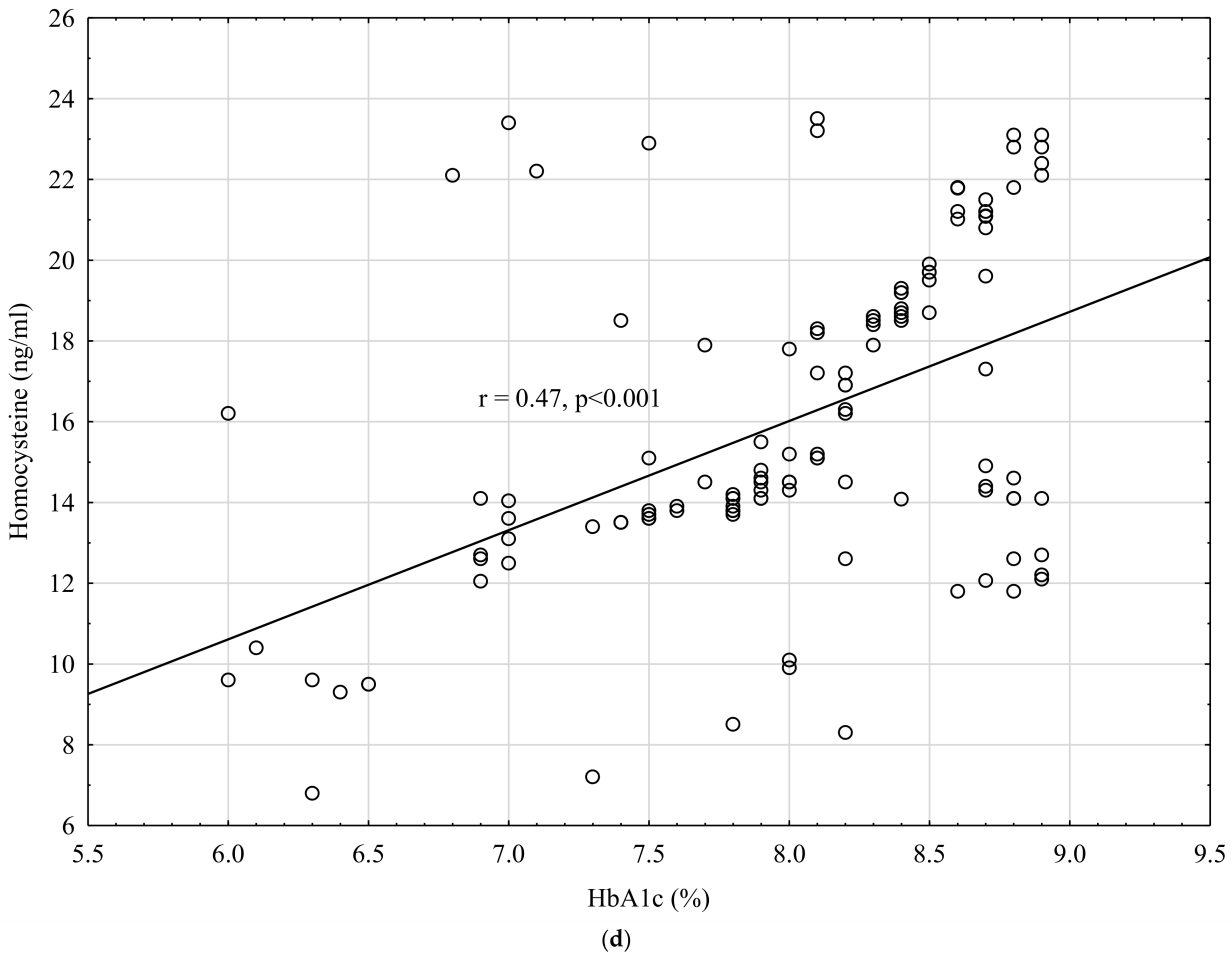

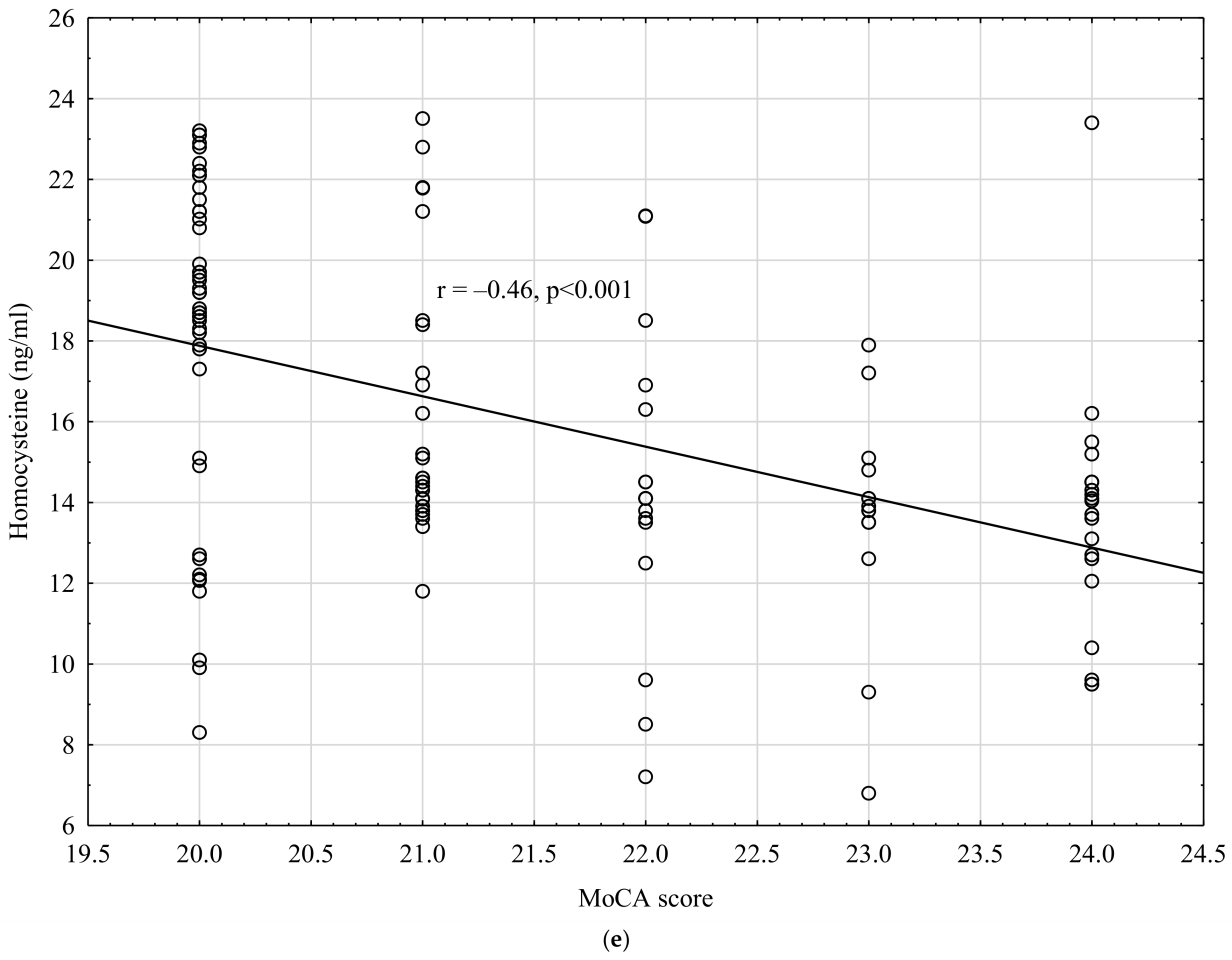
Significant associations in the elderly T2DM patients with mild cognitive impairment (MCI): (**a**)—Vitamin B12 (pg/mL) and Homocysteine (ng/mL) (**b**)—Vitamin B12 (pg/mL) and HbA_1c_ levels (%); (**c**)—Vitamin B12 (pg/mL) and MoCA score; (**d**)—Homocysteine (ng/mL) and HbA_1c_ levels (%), (**e**)—Homocysteine (ng/mL) and MoCA score; r-correlation coefficient and *p*-significance.

Serum levels of vitamin B12 were significantly decreased in patients with MCI (251 ± 36.9 pg/mL) compared to normal (339.9 ± 40.7 pg/mL, *p* < 0.001) (Figure 1a). As expected, in this group vitamin B12 levels were negatively correlated with HbA_1c_ levels (r = −0.53, *p* < 0.001) (Figure 2b), FBG (r = −0.52, *p* < 0.001, and positively with MoCA score (r = 0.65, *p* < 0.001) (Figure 2c). Also, a positive but weak correlation was found between vitamin B12 levels and years of education (r = 0.19, *p* = 0.03), and a negative association with duration of T2DM (r = −0.26, *p* = 0.003).

Serum levels of homocysteine were significantly increased in individuals with MCI (15.9 ± 4 ng/mL [0.118 ± 0.03 μmol/L]) compared to cognitively normal diabetic patients (11.4 ± 3.1 ng/mL [0.084 ± 0.023 μmol/L], *p* < 0.001) (Figure 1b) and inversely correlated with vitamin B12 levels (r = −0.62, *p* < 0.001) (Figure 2a) and with MoCA scores (r = −0.46, *p* < 0.001) (Figure 2e). Additionally, homocysteine levels were positively correlated with HbA_1c_ levels (r = 0.47, *p* < 0.001) (Figure 2d), FBG (r = 0.4, *p* < 0.001), duration of T2DM (r = 0.4, *p* < 0.001), and the number of comorbidities (r = 0.22, *p* = 0.01). The matrix correlation is shown in Table 2.

### 3.3. Logistic Regression Models

The univariate logistic regression analyses indicated that factors that increased the likehood of MCI diagnosis in elderly patients with type 2 diabetes were: older age and fewer years of formal education, longer history of diabetes, a higher number of co-morbidities, higher levels of HbA_1c_, triglycerides and homocysteine, lower levels of vitamin B12, presence of CVD, hypertension, hyperlipidemia, retinopathy, and nephropathy (Table 3). Finally, a multivariate logistic regression model was developed to identify the predictors of MCI. Independent factors associated with MCI included: lower levels of vitamin B12, higher levels of triglycerides, increased number of co-morbidities and fewer years of formal education (Table 4).

## 4. Discussion

The study aimed to determine vitamin B12 and homocysteine levels in older patients with T2DM, both with and without cognitive impairment. It was first found that serum levels of vitamin B12 were significantly decreased in patients with MCI compared to cognitively normal, and inversely correlated with homocysteine concentrations. Whereas levels of homocysteine were observed to be significantly higher in subjects with cognitive impairment compared to cognitively normal. There are several studies describing the association between vitamin B12 or homocysteine and cognitive health in the general population [15,20,22,28,29]. In a cross-sectional study that enrolled 1773 older adults, the authors showed that low levels of vitamin B12 were associated with lower cognitive status assessed using the Mini-Mental State Examination (MMSE) [28]. Also, in an analysis that included a large sample of community-dwelling individuals aged 60–85 years, the researchers indicated that low vitamin B12 and higher concentrations of homocysteine contributed to poorer cognitive performance [20]. In another study based on the NHANES database, a U-shaped association was identified, where low or high levels of vitamin B12 can increase the risk of cognitive impairment [29]. The harmful effects of high concentrations of vitamin B12 are reflected by specific coexisting metabolic and renal diseases such as kidney stones, hypertension, overweight, and hyperuricemia [29]. On the other hand, a systematic literature review revealed that treatment with vitamins B_6_, B_12_ and/or folic acid led to a significant decrease in homocysteine levels in patients with MCI, considering that hyperhomocysteinemia is a known risk factor for cognitive decline [30]. Studies that analyzed the association of vitamin B12 or homocysteine in patients with cognitive impairment in the diabetic population do not provide sufficient results. In a research study, the authors examined the cognitive status of community-dwelling older individuals (including diabetics) and found that vitamin B12 had no association with any cognitive test [31]. However, the study revealed important risk factors for cognitive decline in older adults, such as glycated hemoglobin, which was inversely correlated to processing speed and executive function, as well as geriatric depression, which correlated inversely with visuospatial abilities [31]. In our study, higher levels of HbA_1c_ were one of the independent factors increasing the likelihood of diagnosis of MCI in univariate analysis. On the other hand, both HbA_1c_ and vitamin B12 levels can be influenced by duration of diabetes. Hence, we took into account that variable in the univariate regression analysis, and confirmed that, among other factors, a longer history of diabetes increased the likelihood of MCI diagnosis in elderly patients with type 2 diabetes. However, we did not observe this effect in the multivariate model. In our study, HbA_1c_ showed a strong univariate association with MCI, but was not retained in the final multivariate model. This is likely due to the complex relationship between glucose metabolism, vitamin B12 levels, and the overall comorbidity burden. The overlapping variance among these clinical factors indicates that their effects are highly interrelated in patients with MCI. In another study, elderly diabetic patients with mild B12 deficiency received supplementation [32]. The results showed no improvement in cognitive decline; however, they revealed an increase in serum active B12 and a notable decrease in serum homocysteine levels after 27 months of the intervention [32]. Conflicting data from previously mentioned studies and the observational aspect of our investigation do not support a suggestion for medical treatment. The use of vitamin B12 supplements as a preventive approach must be viewed carefully, acknowledging the necessity for effectively designed future studies. A retrospective study showed that hyperhomocysteinemia is a risk factor for cognitive impairment in adults with T2DM [33]. At the same time, contradictory results were obtained in a small group of 97 diabetic patients, where the final model of logistic regression revealed no association between serum homocysteine and cognitive function [34].

As mentioned above, in our study, patients with cognitive impairment had significantly lower levels of vitamin B12 and increased concentrations of homocysteine compared to cognitively normal individuals. However, the mechanism that could explain how these factors contribute to cognitive health has not yet been fully identified. Firstly, both vitamin B12 deficiency [35,36] and hyperhomocysteinemia [37] have been shown to lead to brain atrophy, although studies on supplementation with vitamin B12 provided contradictory results. A research study showed that the treatment had no beneficial effect on brain volumes [37]. Another study revealed that supplementation with B-vitamins (including B12, B6, and folic acid) caused a decrease in overall brain atrophy and loss of gray matter, thereby slowing cognitive decline in MCI subjects [38]. Secondly, hyperhomocysteinemia, being a consequence of deficiency of vitamin B12, is a common observation in the literature, constituting a well- known risk factor for diabetic complications [39]. The underlying pathophysiological processes leading to these complications include the involvement of homocysteine in increasing oxidative stress, causing inflammation, promoting thrombosis, disrupting endothelial function, and enhancing cell proliferation [40]. Concerning all these mechanistic interpretations linking vitamin B12 and homocysteine to diabetes and cognitive decline, we emphasize that these pathways have not been evaluated in the current research. They should be presented more clearly as theoretical mechanisms rather than explanations supported by available data.

Our study found a strong inverse correlation between levels of vitamin B12 and homocysteine. It was observed that subjects with MCI were more often diagnosed with CVD, hypertension, hyperlipidemia, retinopathy and nephropathy. Besides, homocysteine as a neurotoxin can contribute to the development of cognitive impairment or dementia independently of the impact on the vascular mechanism. In that context, the results of our study are consistent and show a negative correlation between MoCA score and homocysteine levels, and a positive association between MoCA score and concentration of vitamin B12. Interestingly, while univariate analysis showed a significant association of age and homocysteine with MCI, both factors lost relevance in the multivariate model. Instead, lower levels of vitamin B12, higher levels of triglycerides, increased number of co-morbidities and fewer years of formal education emerged as the only independent statistically significant predictors of MCI. This result indicates that while age and homocysteine are significant factors associated with cognitive impairment, their effects are not independent when adjusted for other confounding variables. Moreover, we recognized that the average educational level (9.5 years) in the MCI group could potentially overestimate MCI prevalence when using the MoCA scale. However, the risk was minimized by using a multi-step diagnostics approach validated for the Polish population [41]. Additionally, the participants were also verified with ADL scales; their classification reflects genuine cognitive impairment rather than educational background. In our study, other factors such as nephropathy, which showed an association with MCI in the univariate analysis, lost their significance in the multivariate model. To eliminate statistical overlap and multicollinearity, kidney parameters like eGFR or serum creatinine were excluded to prioritize this clinical variable. However, because subclinical renal impairment independently drives homocysteine accumulation, the lack of eGFR calls for careful biological assessment concerning vitamin B12 deficiency.

In the present study, the authors found an inverse relationship between vitamin B12 and HbA_1c_ as well as fasting blood glucose, and a positive correlation between homocysteine and glycated hemoglobin or fasting blood glucose. The results are in line with data reported in a recently published study [42]. The authors revealed that subjects with poor glycemic control (HbA_1c_ ≥ 7%) had significantly lower levels of vitamin B12 and showed dyslipidemia and impaired renal function. In our study, higher levels of triglycerides remained an important risk factor for MCI in the multivariate analysis.

Our study did not find any significant differences between the groups in terms of the type of treatment; however, it was shown that patients with MCI were more likely to undergo therapy or receive metformin. Metformin is a known risk factor for B12 deficiency, as prolonged therapy affects absorption of the vitamin [43]. The mechanism includes a disruption of calcium-dependent membrane activity in the ileum, the main location for the absorption of vitamin B12. These discrepancies may be analyzed based on the assessment of metformin doses and time of the treatment, which was not the primary aim of the study. Conversely, a large review summarized the positive influence of metformin use for the development of dementia in diabetes, which supports its great value in therapy [43].

Lastly, the authors showed that patients diagnosed with MCI were significantly older, had a longer duration of diabetes, and a higher number of comorbidities. The common risk factors are consistent with other studies [44,45]. Collectively, the data highlight that cognitive impairment shows a set of interrelated disorders including micronutrient deficiencies, comorbidities, poor glycemic control, microvascular complications, and a progressing aging process. This underlines the importance of a diverse, comprehensive management strategy.

Our study provides a significant contribution to the topic, as so far no research has been conducted among elderly diabetic patients with and without cognitive impairment. It was found that serum levels of vitamin B12 were significantly decreased in patients with MCI compared to cognitively normal individuals and inversely correlated with homocysteine concentrations. Moreover, a significant correlation was identified between these factors and HbA_1c_ or MoCA score in MCI subjects. Finally, the study focused on the crucial risk factor of MCI in the diabetic elderly population, which was confirmed in the multivariate analysis.

The study also has weaknesses. Due to its current cross-sectional design, it was not possible to identify causal relationships between the studied variables and impaired cognitive function. Consequently, a formal mediation pathway cannot be established, and vitamin B12 and homocysteine must be interpreted strictly as contemporaneous, associated biomarkers rather than causal mediators. Our study is also limited because we used backward variable selection. This method excluded important factors like age, diabetes duration, HbA_1c_, and vascular complications. As a result, our model might overstate the role of vitamin B12. The study was conducted in a single center, which may limit the generalizability of the results to other populations. In addition, the strict exclusion criteria required to focus on vitamin B12 metabolism resulted in a highly selected cohort. Although this method enhanced internal validity by eliminating significant confounding factors, it also limits the external validity of our findings regarding real-world, heterogeneous clinical populations. Furthermore, our study did not involve any genetic testing. Variations in genes regulating homocysteine metabolism, such as the MTHFR gene, may potentially influence the serum levels of homocysteine independently of vitamin B12 status. Acknowledging this as a limitation, future studies should incorporate genetic screening to confirm these findings. Additionally, our study did not account for the exact daily dose and the duration of metformin therapy, which are known to adversely affect the absorption of vitamin B12 and consequently, homocysteine levels. Other longitudinal studies should include precise dosing and treatment duration details to clarify these mechanisms further. Another limitation of this study is that homocysteine was assessed using a research-quality ELISA kit (General Hcy/Homocysteine ELISA kit, E0772Ge, EIAab, Wuhan, China) reporting in ng/mL, rather than standard clinical methods (such as HPLC) that quantify total homocysteine in μmol/L. The obtained values (11–16 ng/mL, equivalent to 0.081–0.118 μmol/L) likely reflect a specific free fraction of homocysteine, which limits direct comparison with established clinical thresholds for hyperhomocysteinemia. Furthermore, the lack of measurement of serum folate and methylmalonic acid (MMA) prevents the detection of deficiency at the tissue level. Lastly, the study did not analyze data on eating habits or dietary intake of vitamin B12 that could have an important effect on both the level of the vitamin and cognitive impact.

## 5. Conclusions

In summary, our findings highlighted significant associations of lower levels of vitamin B12, higher concentrations of triglycerides, increased number of comorbidities and fewer years of formal education with the diagnosis of MCI in elderly patients with T2DM. Importantly, although homocysteine demonstrated a significant relation in the univariate analysis, its influence was diminished in the final multivariable model. The results emphasize the importance of regular screening of vitamin B12 levels as well as routine cognition tests in diabetic elderly subjects. Further longitudinal cohort studies and randomized controlled trials are warranted to clarify the temporal sequence and mechanisms underlying the association between diabetes and cognitive impairment.

## Figures and Tables

**Table 1:** Clinical and laboratory characteristics of T2DM patients, comparing subjects with mild cognitive impairment (MCI) and those without MCI (Normal).

	Mild Cognitive Impairment (n = 126)		Normal (n = 259)		Statistics	*p* Value
	Mean	SD	Mean	SD	Z/t	
Continuous variables						
Age (years) *	74.6	3.9	72.1	3.7	−6.1	<0.001
Education (years) *	9.5	1.8	11.8	2.4	8.9	<0.001
T2DM duration (years) *	10.7	6.2	6.5	5.2	−7.9	<0.001
BMI (kg/m^2^)	29.6	3.5	29.3	3.5	−1.1	0.26
HbA_1c_ (%) *	7.9	0.7	7.1	0.6	−10.2	<0.001
Total-C (mmol/L) *	4.83	0.95	4.67	0.95	−2.5	0.01
LDL (mmol/L)	2.78	0.72	2.72	0.74	−1.4	0.15
HDL (mmol/L) *	1.15	0.29	1.19	0.21	2.4	0.02
TG (mmol/L) *	2.04	0.52	1.92	0.36	−4.3	<0.001
FBG (mmol/L)	7.37	1.5	7.18	1.29	−1.23	0.21
SBP (mmHg)	135.5	16.1	135.1	15.1	−0.08	0.93
DBP (mmHg)	75.3	7.7	74.9	7.9	−0.33	0.74
Number of comorbidities *	7.2	3.4	3.7	2.6	−8.7	<0.001
MoCA score *	21.5	1.5	27.2	1.2	15.9	<0.001
Categorical variables	Frequency	%	Frequency	%	χ^2^	*p* Value
Female gender	59	46.8	115	44.4	0.2	0.65
Current smokers	42	33.3	79	30.5	0.32	0.57
No physical activity	62	49.2	128	49.4	0.01	0.97
DM complications						
CVD *	100	79.4	103	39.8	53.3	<0.001
Stroke	6	4.8	10	3.9	0.17	0.68
Hypertension *	114	90.5	208	80.3	6.4	0.01
Hyperlipidemia *	115	91.3	185	71.4	19.4	<0.001
Diabetic polyneuropathy	23	18.3	31	11.9	2.78	0.09
Retinopathy *	94	74.6	115	44.4	31.2	<0.001
Nephropathy *	70	55.5	94	36.3	12.8	0.003
Current treatment						
OAD	121	96	247	95.4	0.09	0.77
Metformin	101	80.2	184	71	3.6	0.06
Insulin	53	42.1	110	42.5	0.01	0.94

Values are presented as mean ± standard deviation (SD) or frequency (%). * Significance, *p* < 0.05; Mann–Whitney U test (Z) and *t* test (*t*) for continuous variables or χ^2^ test for categorical variables were used to test for significant differences between patients with MCI and those without MCI (Normal). Abbreviations: CVD—cardiovascular disease, BMI—body mass index, T2DM—type 2diabetes, Total-C—total cholesterol, FBG—fasting blood glucose, HbA_1c_—glycosylated hemoglobin, HDL—high-density lipoprotein cholesterol, LDL—low-density lipoprotein cholesterol, MCI—mild cognitive impairment, MoCA—Montreal Cognitive Assessment, OAD—oral antidiabetic drugs, TG—triglycerides.

**Table 2:** Correlation analyses among vitamin B12 and homocysteine levels, HbA_1c_, and MoCA score in the studied diabetic patients.

	Vitamin B12 (pg/mL)	Homocysteine (ng/mL)	HbA_1c_ (%)	MoCA Score
All subjects				
Vitamin B12 (pg/mL)	1	−0.81 ***	−0.59 ***	0.69 ***
Homocysteine (ng/mL)		1	0.52 ***	−0.58 ***
HbA_1c_ (%)			1	−0.51 ***
MoCA score				1
Mild Cognitive Impairment (n = 126)				
Vitamin B12 (pg/mL)	1	−0.62 ***	−0.53 ***	0.65 ***
Homocysteine (ng/mL)		1	0.47 ***	−0.46 ***
HbA_1c_ (%)			1	
MoCA score				1
Normal (n = 259)				
Vitamin B12 (pg/mL)	1	−0.79 ***	−0.29 ***	0.24 ***
Homocysteine (ng/mL)		1	0.26 ***	−0.28 ***
HbA_1c_ (%)			1	−0.08
MoCA score				1

Data are presented as r- correlation coefficient, *p*-significance: *** *p* < 0.001. MoCA—Montreal Cognitive Assessment, HbA_1c_—glycosylated hemoglobin.

**Table 3:** Univariate logistic regression analysis of risk factors for mild cognitive impairment (MCI) in T2DM elderly patients.

Parameter	ß	SE of ß	*p* Value	OR	95% CI
Age (years) *	0.162	0.03	<0.001	1.18	1.11–1.25
Education (years) *	−0.55	0.067	<0.001	0.57	0.51–0.66
T2DM duration (years) *	0.129	0.022	<0.001	1.14	1.09–1.19
BMI (kg/m^2^)	0.028	0.031	0.36	1.03	0.96–1.09
HbA_1c_ (%) *	1.81	0.193	<0.001	6.14	4.2–8.94
Total-C (mmol/L)	0.005	0.003	0.1	1.01	0.99–1.01
LDL (mmol/L)	0.002	0.004	0.5	1.01	0.99–1.01
HDL (mmol/L)	−0.02	0.01	0.07	0.98	0.95–1.00
TG (mmol/L) *	0.008	0.003	0.01	1.01	1.01–1.02
FBG (mmol/L)	0.005	0.004	0.22	1.01	0.99–1.01
SBP (mmHg)	0.002	0.007	0.8	1.01	0.98–1.01
DBP (mmHg)	0.005	0.014	0.73	1.01	0.98–1.03
Number of comorbidities *	0.35	0.041	<0.001	1.42	1.31–1.54
Female gender	0.09	0.2	0.65	1.1	0.71–1.69
Current smokers	0.13	0.23	0.57	1.14	0.72–1.79
No physical activity	−0.009	0.21	0.96	0.99	0.65–1.52
CVD *	1.76	0.254	<0.001	5.83	3.54–9.58
Stroke	0.22	0.52	0.67	1.24	0.44–3.51
Hypertension *	0.85	0.34	0.01	2.33	1.19–4.54
Hyperlipidemia *	1.43	0.34	<0.001	4.18	2.13–8.21
Diabetic polyneuropathy	0.49	0.3	0.09	1.64	0.91–2.95
Retinopathy *	1.302	0.24	<0.001	3.68	2.29–5.88
Nephropathy *	0.786	0.221	<0.001	2.19	1.42–3.38
OAD	0.16	0.54	0.76	1.17	0.41–3.41
Metformin	0.499	0.262	0.06	1.65	0.98–2.75
Insulin	−0.02	0.22	0.93	0.98	0.63–1.51
Vitamin B12 (pg/mL) *	−0.048	0.005	<0.001	0.95	0.94–0.96
Homocysteine (ng/mL) *	0.343	0.039	<0.001	1.41	1.31–1.52

* Statistically significant difference, *p* < 0.05; ß: regression coefficient; CI: confidence interval for odds ratio; OR: odds ratio; SE: standard error; Abbreviations: CVD—cardiovascular disease, BMI—body mass index, T2DM—type 2diabetes, Total-C—total cholesterol, FBG—fasting blood glucose, HbA_1c_—glycosylated hemoglobin, HDL—high-density lipoprotein cholesterol, LDL—low density lipoprotein cholesterol, MCI—mild cognitive impairment, OAD—oral antidiabetic drugs, TG—triglycerides.

**Table 4:** Multivariate logistic regression analysis of risk factors for mild cognitive impairment (MCI) in T2DM elderly patients.

Parameter	ß	SE of ß	*p* Value	OR	95% CI
Vitamin B12 (pg/mL) *	−0.05	0.005	<0.001	0.96	0.94–0.97
TG (mmol/L) *	0.02	0.005	0.002	0.98	0.97–0.99
Number of comorbidities *	0.23	0.06	<0.001	1.26	1.11–1.43
Education (years) *	−0.31	0.10	0.002	0.73	0.59–0.89

* Statistically significant difference, *p* < 0.05; ß: regression coefficient; CI: confidence interval for odds ratio; OR: odds ratio; SE: standard error; Abbreviations: TG—triglycerides.

## Data Availability

The data presented in this study are available on request from the corresponding author.
